# Adenomyosis or Not: The Ambiguity of Diagnostic Results and Clinician Incertitude

**DOI:** 10.7759/cureus.17902

**Published:** 2021-09-12

**Authors:** Fatimah Rajabally, Rama Alkhaldi, Hassenjee Joomye

**Affiliations:** 1 Department of Medicine, Royal College of Surgeons in Ireland, Dublin, IRL; 2 Department of Radiology, Clinique Darné, Floréal, MUS

**Keywords:** gynaecology and obstetrics, adenomyosis, female reproductive health, gynaecological endoscopy, mri

## Abstract

Adenomyosis is a benign gynecological condition caused by the presence of the endometrial glands within the uterine walls. This phenomenon occurs due to the breakdown of the inner lining of the uterus (endometrium) through the muscle wall of the uterus (myometrium). Usual symptoms are pelvic pain and irregular vaginal bleeding. As it is defined according to histological criteria obtained from a hysterectomy, diagnosis made based on only symptoms and imaging can be challenging. Discussed here is a case of a 30-year-old woman who presented with severe pelvic pain and irregular periods. Computing imaging (CT) and blood tests initially suggested malignancy but a review of magnetic resonance imaging (MRI) scans finally concluded adenomyosis. This clinical scenario elucidates for the utmost caution in the interpretation of investigations especially for the growing number of young women with this condition urging for more accurate diagnostic tools and effective communication between clinicians.

## Introduction

Uterine adenomyosis is a gynecological disease in which the endometrial glands and stroma are present within the myometrium, causing hypertrophy of the surrounding myometrium. The condition has more recently been discovered in young fertile women due to improvements in diagnostic tools. Women with symptomatic adenomyosis may display uterine enlargement, abnormal uterine bleeding (AUB), infertility, and/or painful menses negatively impacting their quality of life [1]. The definite diagnosis of this disorder is made from histological examinations of uterine segments obtained from a hysterectomy. This poses a challenge to young women who want to conceive as a hysterectomy is a major surgery causing infertility [2]. Pelvic imaging through ultrasound and MRI can also detect signs of the condition; nevertheless, the diagnostic process remains difficult due to variances in the definition and classification of adenomyotic lesions from both the histopathology and the imaging point of view. The pathogenesis and the different phenotypes of the disease are poorly understood. The disorder can also coexist with other gynecological conditions such as endometriosis and uterine leiomyomas increasing the variability of the available data [1]. 

## Case presentation

A 30-year-old female presented to her general practitioner (GP) with a six-month history of severe non-cyclic pelvic pain, heavy periods, and bloating. The patient’s menarche was at 14, and subsequently, she had regular cycles. However, for the past couple of months, she has been experiencing irregular periods. She had never used any form of oral contraception and did not have any record of previous medical, obstetrical, or surgical history.

Her symptoms were intermittent for the first five months and progressively became permanent. The pain originated from the lower abdomen and radiated to the lumbar area. She described the pain as dull and pulsating, feeling “two pinpoint pressures at the sides,” which indicated bilateral pelvic pain. Her discomfort intensified at night prompting the use of paracetamol to ease the pain. Her GP prescribed her progesterone tablets for the irregular menses and referred her to a specialist ordering blood tests, a pelvic ultrasound scan, and a CT scan. The radiologist noted on both the ultrasound and CT scan an enlarged uterus with endometrial hyperplasia (endometrium thickness with cystic areas) which he queried as being of possible malignant origin. 

Faced with these results, the gynecologist, decided to perform a hysteroscopy and a dilation and curettage (D&C). Pre-operative blood tests results revealed anemia with hemoglobin (Hb) at 8.4g/dL. During the procedure, some areas of endometrial hyperplasia were seen but no other intra-uterine abnormalities were noted. The histopathologic results of the D&C, however, showed benign tissue. After the operation, the patient was prescribed non-steroidal anti-inflammatory drugs (NSAIDs) for the pain and was recommended to continue taking paracetamol along with the progesterone tablets for her irregular periods. Due to the lockdown restrictions imposed by the current pandemic, the patient did not turn up for follow-ups post-surgery. 

**Figure 1:**
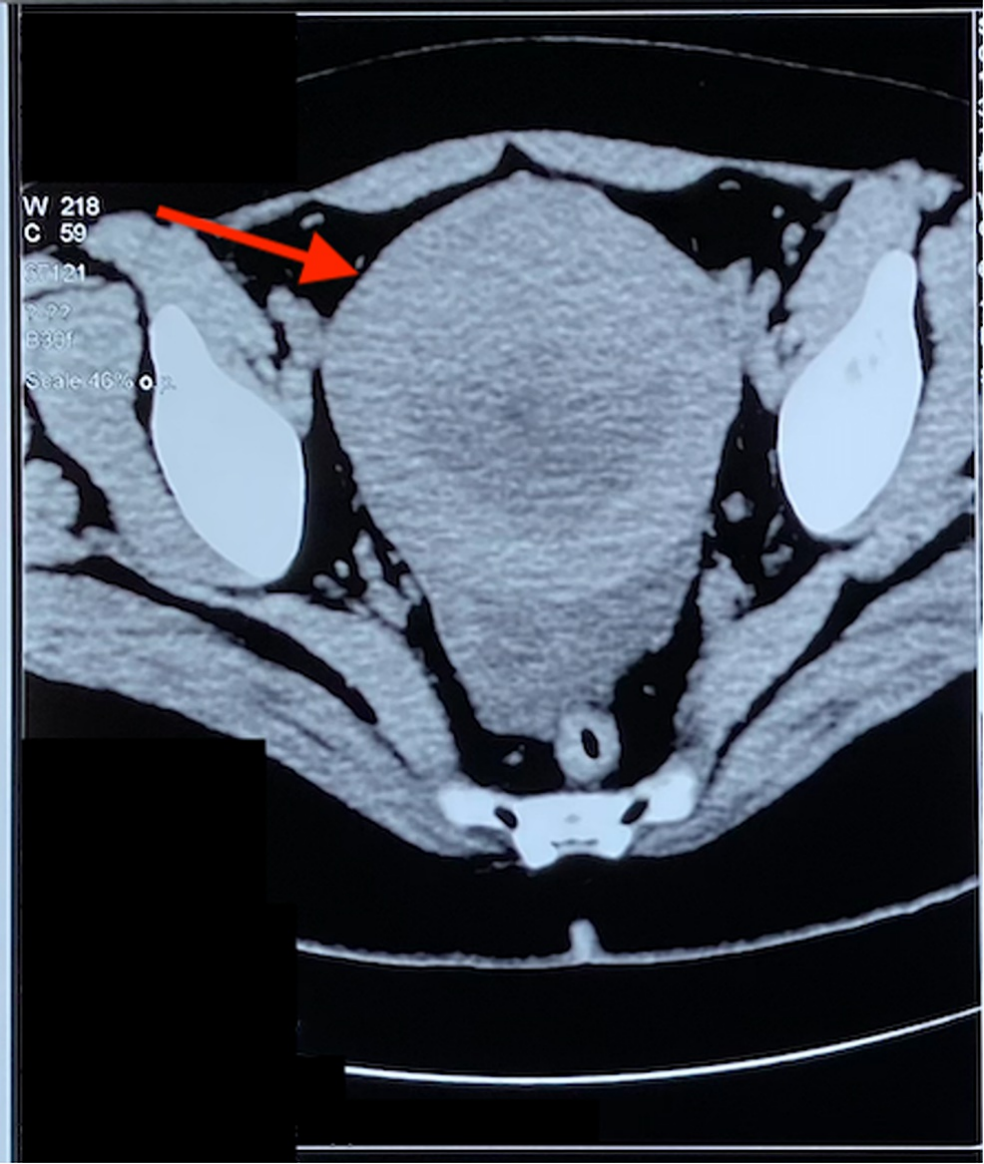
Axial CT scan of pelvis demonstrating an enlarged uterus of 113 mm/ 105 mm/ 135 mm. CT: computed tomography

Two months later, the patient came back to the gynecologist for a consult as the pain worsened. During her physical examination, it was noted that her uterus had become palpable abdominally. A second set of tests, this time including an MRI scan, was ordered and done by another radiologist revealing an enlarged uterus in the CT scan (Figure 1).

It was noted again that there was a centro-uterine lesion with endometrial hyperplasia; this radiologist also raised the possibility of malignancy but gave a differential diagnosis of necrosis of a submucosal fibroid. Bilateral ovarian cysts of 4 cm were also noted. Blood test results on this occasion showed elevated cancer antigen 125 (CA-125) levels at 285.5 u/ml. 

Consequently, given the discrepancies between the two radiologists’ reports, the patient self-referred herself to another gynecologist who decided to perform yet another hysteroscopy and D&C. The hysteroscopy failed to reveal any new findings and histopathology was again in favor of benign hyperplasia (complex hyperplasia without atypia). 

**Figure 2:**
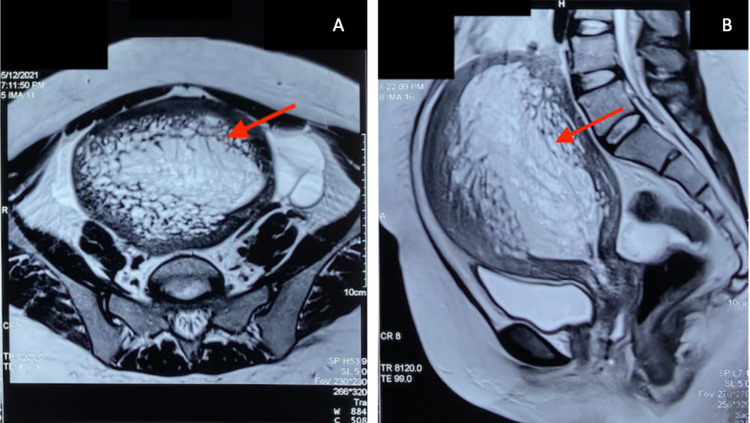
T2-weighted MRI scans revealing adenomyosis seen as enlarged junctional zone with indistinct margins infiltrating into the myometrium covered with cystic areas (arrowheads) (A) axial view (B) sagittal view. MRI: magnetic resonance imaging

The second gynecologist submitted this case to a multidisciplinary team compromising of an oncologist, radiologist, and pathologist who had not yet been involved in the previous investigations. A careful review of the MRI scans revealed the disappearance of the endometrial lining and the presence of small cystic areas in both the axial (Figure 2A) and sagittal (Figure 2B) planes. This led to the conclusion of an atypical and severe form of adenomyosis.

Due to the patient’s reproductive age, the patient was prescribed Gonadotrophin Releasing Hormones (GnRH) analogs and is now much better. After two monthly injections, she described a net improvement in her symptoms with much less pain and reduced menses. 

## Discussion

What presented as a common complaint for a woman, turned out to be a more complex issue due to the initial difficulty in reaching a diagnosis after imaging and two diagnostic D&Cs. Further confusion arose when attention was given to the presence of bilateral ovarian cysts and raised CA-125 levels. CA-125 is a tumor marker that can be elevated in cases of serous cell carcinoma of the ovary, but high levels may also be found in benign conditions such as adenomyosis, endometriosis, fibroids, or pelvic inflammations, emphasizing its non-specificity. Discussion within the multidisciplinary team after reviewing the scans finally led to the conclusion that the bilateral ovarian cysts were benign in nature and that the elevated CA-125 level was most probably due to the presence of adenomyosis. 

Risk of Ovarian Malignancy Algorithm (ROMA) is a diagnostic tool increasingly being used to detect certain gynecological conditions eradicating the need for redundant procedures. It is a qualitative serum test that derives a numerical score from the results of CA-125 and human epididymis protein 4 (HE4) blood tests in addition to menopausal status, to identify patients presenting with an adnexal mass as having malignancy [3]. In this case, it was not used but it could have helped in speeding up the differential diagnosis. 

This case also highlights how the lack of communication and collaboration between doctors hinders the progress towards a diagnosis. Adenomyosis is more commonly seen in women over the age of 40, with a history of prior cesarean section and/or uterine surgery, and who are multiparous [1]. None of these criteria fit the patient, and there was a collective failure in the diagnostic interpretation by clinicians to correlate the radiological findings with the patient’s history. The CT scans, mainly utilized for identifying malignancies, were misleading since it was initially thought to be endometrial hyperplasia. A pelvic ultrasound was also conducted, but its use for the diagnosis of adenomyosis is difficult. The Morphological Uterus Sonographic Assessment (MUSA) group created a set of guidelines to aid in the diagnosis of the myometrium and uterine masses, yet it is still unclear what ultrasound features specifically suggest adenomyosis [4]. In this case, the key imaging findings leading to the diagnosis were from the MRI where the clear disappearance of the endometrial lining was indicative of adenomyosis. A confirmed diagnosis of this condition is typically made with a histopathological sample obtained from a hysterectomy; however, this was not an option due to the patient’s young age and reproductive status.

Ultimately, this condition requires a long-term management plan involving medical and/or surgical treatment. Treatment for adenomyosis is dependent on factors such as the woman’s age, reproductive status, and clinical symptoms. Due to potential risks affecting the option to conceive, conservative surgical approaches involve hysteroscopic endometrial and adenomyoma resection, endometrial ablation, high-intensity focused ultrasonography (HIFU), laparoscopic resection of adenomyosis, or uterine artery embolization. However, these treatments require further corroboration due to a lack of supporting evidence [1]. 

## Conclusions

Despite significant advancements and understandings in diagnostic techniques, potential medical treatments, and pathogenic mechanisms, a pressing need for a consistent diagnostic criteria profile of imaging and histologic examinations is required to accurately recognize all the variations in phenotypes of adenomyosis. This case study highlights the faulty interpretation of diagnostic investigations and lack of cooperation between physicians lowering the quality of care for the patient, with delay in diagnosis and appropriate treatment.
